# Case report: Open-heart removal for a cement embolism formed 10 years ago in the right ventricle and pulmonary artery

**DOI:** 10.3389/fcvm.2023.1221525

**Published:** 2023-07-18

**Authors:** XinPei Liu, PeiShan Chu, Qi Miao

**Affiliations:** Department of Cardiac Surgery, Peking Union Medical College Hospital, Beijing, China

**Keywords:** cement embolism, pulmonary artery, case report, surgery, right ventricle

## Abstract

Bone cement embolism is a known complication after a kyphoplasty operation. Cement embolisms without immediate fatal complication such as cardiac perforation or hypoxemia were often stable during observation. We report a case of a large volume bone cement embolism involving the right ventricle and the pulmonary artery system. The patient developed mild exertional shortness of breath and chest pain after a percutaneous kyphoplasty (PKP) operation 10 years ago. However, her mild symptoms were attributed to multiple myeloma, and no chest imaging was taken until the symptoms exacerbated after a COVID-19 infection 6 months ago. A large, tree-branch-shaped embolus was found, causing severe obstruction of the ascending and middle-lobe branch of the right pulmonary artery. The pulmonary perfusion scintigraphy demonstrated an impaired perfusion of the right upper and middle lobe. An open-heart removal was performed, and the symptoms were relieved afterward. We report this case to highlight the importance of routine chest imaging after a PKP operation and to claim that open-heart removal for chronic cement pulmonary embolism is technically feasible and safe.

## Introduction

Bone cement embolization is a well-known complication after a percutaneous kyphoplasty (PKP) operation. Severe cement embolism can cause life-threatening cardiac perforation and need percutaneous retrieval or open-heart surgery immediately after the PKP operation (1, 2). For patients without immediate life-threatening symptoms, a conservative observation is often recommended (3). Cement embolisms were often stable during observation. Late occurrences of symptoms that need open-heart surgery were seldom reported in the literature.

## Case description

**Figure 1:**
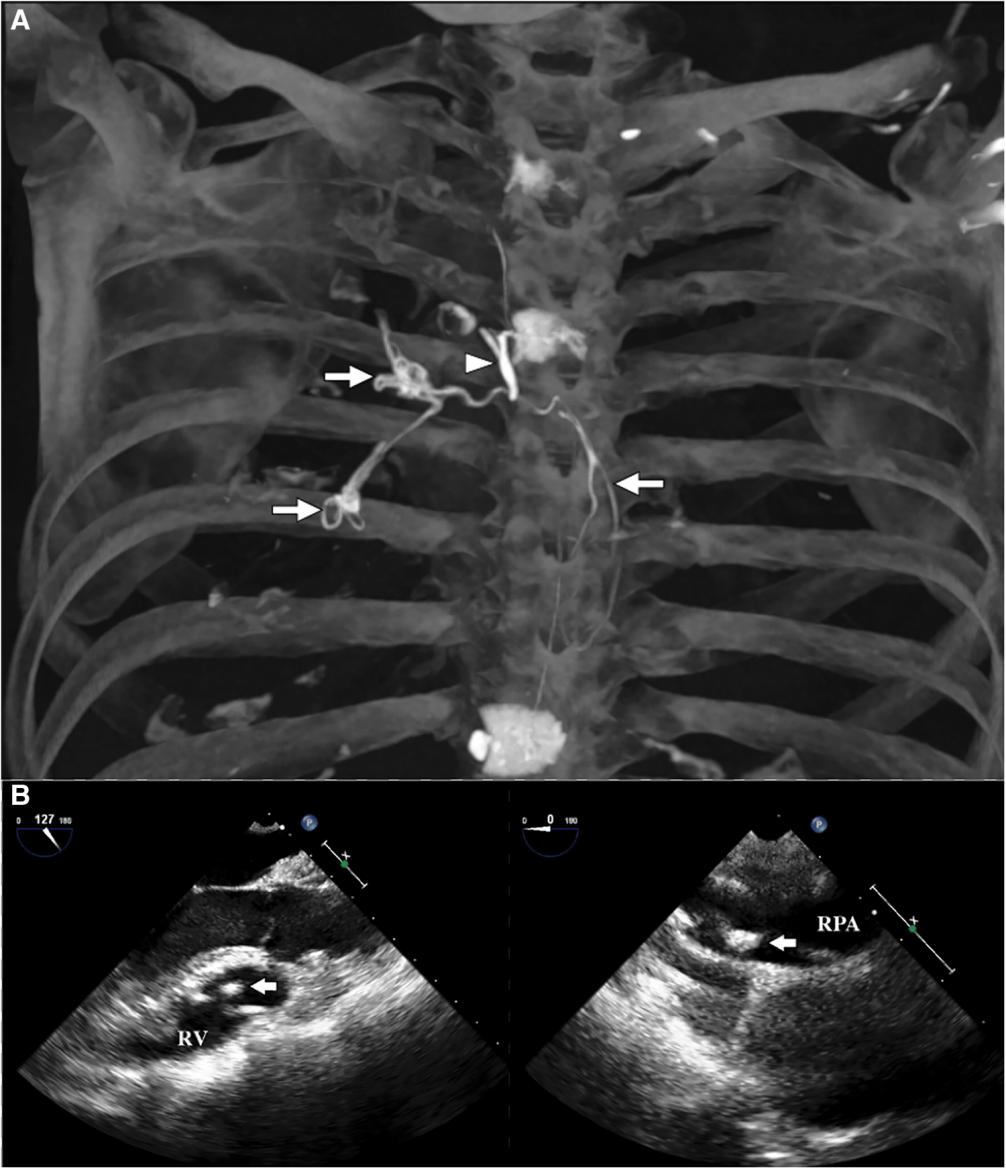
(**A**) The three-dimensional CT reconstruction demonstrated a tree-branch-shaped high-density foreign material in the RV (long arrow), extending through the MPA and RPA into the ascending branch and middle-lobe branch of the RPA (long arrow), indicating the diagnosis of PMMA cement embolism. The cement seemed to enter the vein system through the azygos vein (**short arrow**). (**B**) The transesophageal echocardiography demonstrated a rod-shaped hyperechoic foreign body in the RV and RPA (arrow).

**Figure 2:**
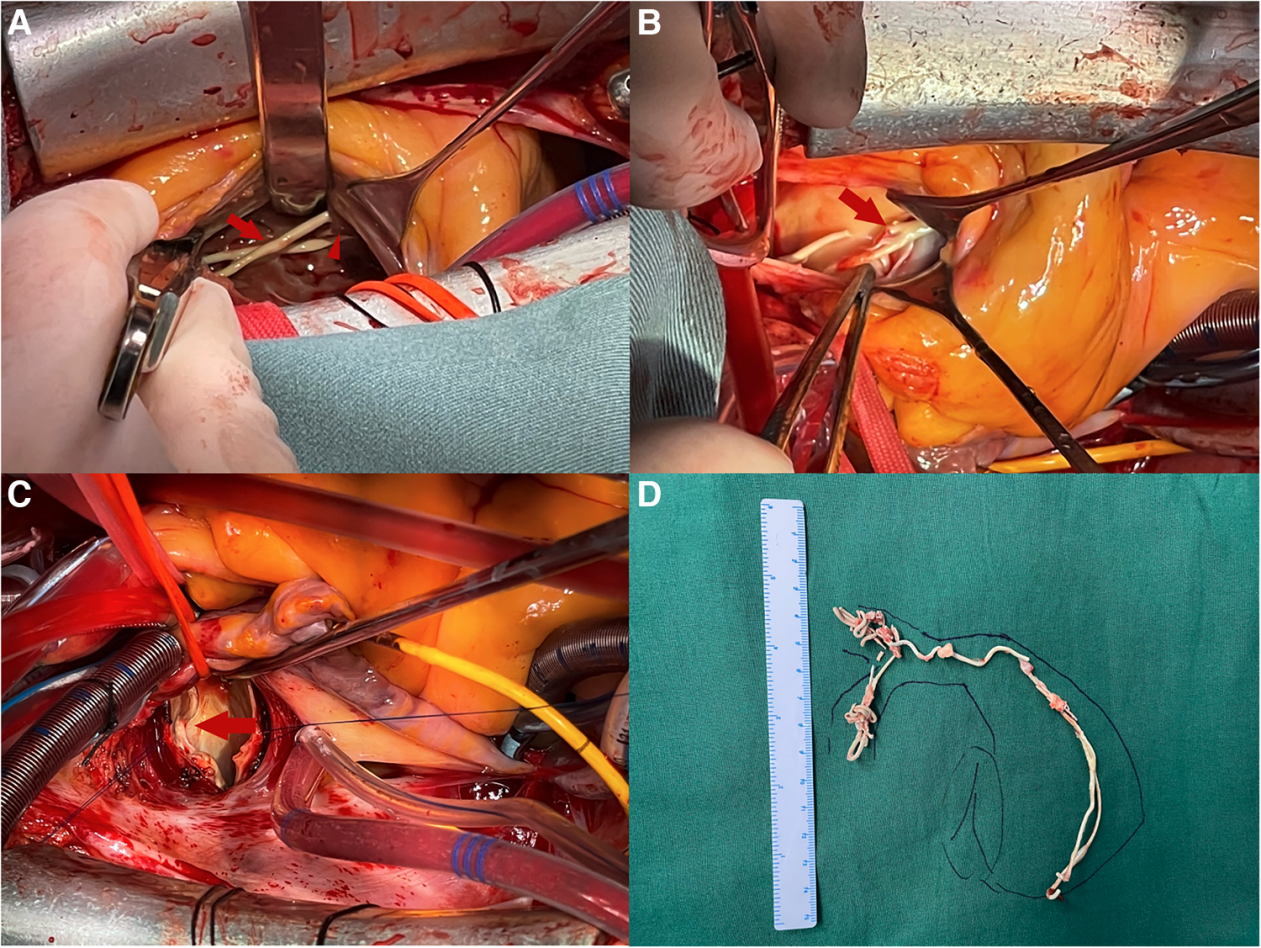
(**A**) Through an RA incision, a circumferential piece of the PMMA cement (long arrow) can be seen tangling with tricuspid chorda (short arrow). (**B**) Through an MPA incision, the PMMA cement was found to be fractured with a sharp end. (**C**) The PMMA cement (arrow) can be seen through an RPA incision. (**D**) The gross appearance of the surgically resected specimen, indicating that the PMMA cement embolus was completely removed.

A 52-year-old woman was referred to our hospital for further workup and treatment of a high-density foreign body in her right ventricle (RV) and pulmonary artery (PA). She had a history of PKP operation for multiple myeloma 10 years ago. She recalled no discomfort immediately after the operation; thus, no postoperative imaging was taken. She developed mild exertional shortness of breath and chest pain since then. However, those symptoms were explained as bone pain and were attributed to multiple myeloma by her primary care physician. Six months ago, after a COVID-19 infection, her symptoms exacerbated, and a chest CT scan was taken. The CT scan found a high-density material in her RV, main pulmonary artery (MPA), and right pulmonary artery (RPA). A three-dimensional reconstruction based on a computed tomography for pulmonary artery demonstrated a tree-branch-shaped foreign body in the RV, extending through the MPA and RPA into the right upper and middle-lobe pulmonary artery (Figure 1A). The intertwined cement embolus obstructed the ascending branch and the middle-lobe branch of the RPA, and the right apical pulmonary artery was totally occluded. The transesophageal echocardiography found a rod-shaped hyperechoic foreign body in the RV and RPA (Figure 1B) and 2 + pulmonary regurgitation. The pulmonary artery pressure was normal. The patient was therefore diagnosed with polymethylmethacrylate (PMMA) embolism to the RV and PA. The pulmonary perfusion scintigraphy demonstrated an impaired perfusion of the right upper and middle lobe. Her 6-min walking distance was 370 m preoperatively. We assumed the symptoms to be explained by the embolism. Due to its large volume and complex shape, endovascular retrieval of the PMMA cement was not considered, and an open-heart surgery was scheduled.

The details of the surgical operation was shown in the Supplementary Video. The RV was explored through a right atrial incision, and a circumferential cement was found entangled with a tricuspid chorda (Figure 2A). We freed the chorda by cutting the cement apart. Through an MPA incision, the cement was found to be fractured with a spear-shaped end (Figure 2B). Therefore, the ventricle part of the cement was removed. Then an RPA incision was made, and the body temperature was decreased to 18°C. The cement in the ascending and middle-lobe branch was removed from the intima by meticulous blunt dissection (Figure 2C), under transient circulatory arrest (5 min for each branch). The PMMA embolism was completely removed (Figure 2D). The cardiopulmonary bypass (CPB) was weaned uneventfully. The total CPB time was 108 min. The patient was extubated on the first postoperative day and discharged 5 days later. One month after the surgery, the patient reported an uneventful recovery, without chest pain or shortness of breath. Her 6-min walking distance increased to 700 m. A pulmonary perfusion scintigraphy review was recommended but refused by the patient.

## Comments

Since introduced in the 1980s, the percutaneous kyphoplasty operation was commonly used in the diagnosis and treatment of vertebral compression fracture and pathologic fracture. An embolism of injected cement to the right heart system and pulmonary arteries is a not-so-rare complication of this operation, with a morbidity of 10% according to literature (3–6). Most small volume cement embolisms are asymptomatic and can be detected by a chest CT scan, while large volume cement embolisms can cause refractory hypoxemia and intraoperative death (7) or RV perforation leading to acute or chronic pericardial tamponade (1, 2). Besides, PMMA cement embolism can also cause tricuspid regurgitation and heart failure (8) and need to be retrieved. Several salvage or emergent surgeries for life-threatening complications due to cement embolism were reported previously. Schoechlin et al. also reported a case of right ventricular perforation that occurred 1 month after a kyphoplasty operation (9). We report the case of a silent cement embolism with chronically progressing symptoms and underwent open-heart surgery 10 years afterward. It was obvious that the PMMA cement had entered her RV system and caused the obstruction during the PKP operation. However, her symptom of mild chest pain after the PKP operation was misdiagnosed as bone pain of multiple myeloma. It took 10 years and a COVID-19 infection that exacerbated the symptom before the large cement embolism was finally detected. An impaired 6-min walking distance and the preoperative pulmonary perfusion scintigraphy can confirm the relationship between her symptoms and the embolism. Moreover, as seen in the video, the cement in the MPA had a sharp break-end, which was possible to cause a perforation during any single heartbeat, leading to what Schoechlin et al. reported (9). Therefore, an open-heart surgery was necessary. This case reminds us that large volume cement embolism can be nearly asymptomatic in the early postoperative period and cause consistent damage, which highlights the importance of postoperative chest imaging.

Several successful endovascular extractions of cement embolism were reported (8). However, due to its large volume and complex shape, the cement embolus of our patient was considered impossible to be retrieved percutaneously. Intraoperatively, it was impressive that the cement could be removed by a careful blunt dissection, and an endarterectomy was not necessary. Therefore, the integrity of intima was kept, and the risk of pulmonary hemorrhage was low. A transient circulatory arrest or low flow perfusion will be very helpful for an excellent surgical view, especially when dealing with distal pulmonary arteries.

## Conclusion

By introducing this clinical case, we aim to highlight the importance of routine chest imaging after a PKP operation and to claim that open-heart removal for chronic cement pulmonary embolism is technically feasible and safe.

## Data Availability

The original contributions presented in the study are included in the article/Supplementary Material, further inquiries can be directed to the corresponding author.
